# Heterozygous loss of Rbm24 in the adult mouse heart increases sarcomere slack length but does not affect function

**DOI:** 10.1038/s41598-020-64667-0

**Published:** 2020-05-06

**Authors:** N. E. de Groot, M. M. G. van den Hoogenhof, A. Najafi, I. van der Made, J. van der Velden, A. Beqqali, Y. M. Pinto, E. E. Creemers

**Affiliations:** 1 Department of Experimental Cardiology, Amsterdam Cardiovascular Sciences, Amsterdam UMC, location AMC, Amsterdam, The Netherlands; 2 Department of Physiology, Amsterdam Cardiovascular Sciences, Amsterdam UMC, location VUmc, Amsterdam, The Netherlands

**Keywords:** Cardiovascular biology, Heart failure

## Abstract

RNA-binding proteins are key regulators of post-transcriptional processes such as alternative splicing and mRNA stabilization. Rbm24 acts as a regulator of alternative splicing in heart and skeletal muscle, and is essential for sarcomere assembly. Homozygous inactivation of Rbm24 in mice disrupts cardiac development and results in embryonic lethality around E12.5. In the present study, we generated somatic Rbm24 knockout (KO) mice and investigated the effects of reduced levels of Rbm24 in the adult heart. Due to the embryonic lethality of Rbm24 KO mice, we examined cardiac structure and function in adult Rbm24 heterozygotes (HETs). Rbm24 protein expression was 40% downregulated in HET hearts compared to WT hearts. Force measurements on isolated membrane-permeabilized myocytes showed increased sarcomere slack length and lower myofilament passive stiffness in adult Rbm24 HET compared to wildtype cardiomyocytes. As a result of the differences in sarcomere slack length, the relations between force development and sarcomere length differed between WT and Rbm24 HET hearts. No differences in sarcomere structure and titin isoform composition were observed. Likewise, *in vivo* cardiac function and myocardial structure was unaltered in Rbm24 HET mice compared to WT, at baseline and upon pressure overload after transverse aortic constriction. In conclusion, we generated a somatic Rbm24 KO model and recapitulated the previously reported embryonic phenotype. In adult Rbm24 HET cardiomyocytes we observed increased sarcomere slack length, but no difference in sarcomere structure and cardiac function.

## Introduction

Alternative pre-mRNA splicing is a fundamental layer of gene expression. It represents the process where exons can be included or excluded in different combinations to create multiple mRNA transcripts from a single pre-mRNA, and as such, it serves to increase the diversity of the transcriptome and proteome. It is estimated that at least 95% of human genes are alternatively spliced and that approximately 100.000 different proteins are produced from 21.000 protein coding genes^1,2^. Alternative splicing is mediated by the spliceosome, a large and dynamic ribonucleoprotein complex composed of proteins and small nuclear RNAs, which recognizes and assembles at splice sites on pre-mRNAs transcripts to cleave the introns and fuse the remaining exons together. The exact make-up of the spliceosome and its association with a range of RNA-binding proteins (RBPs) determines which specific parts of the mRNAs will be included or excluded. Interestingly, numerous RBPs that bind splicing sequences are expressed in a cell-type or tissue-specific manner, allowing the generation of cell-type specific mRNA isoforms^3^.

In the heart, isoform switching of proteins such as titin, troponin T, tropomyosin and myomesin importantly orchestrate the functional properties of the sarcomere^4^. In 2012, Guo and colleagues identified a cardiac splice factor, RNA-binding motif protein 20 (Rbm20) that is responsible for alternative splicing of many cardiac genes^5^. Mutations in this gene cause an arrhythmogenic dilated cardiomyopathy (DCM)^6–9^ and the expression of aberrant titin and CamKIIδ isoforms are considered the main underlying causes for the disease^5,9^. Apart from the identification of Rbm20 mutations as a cause for familial DCM, it has become increasingly clear that numerous biological processes are importantly controlled by splice factors in the heart^10^. For example, the splice factor ASF/SF2 regulates excitation-contraction coupling by alternative splicing of CaMKIIδ^11^; Rbfox1 is involved in hypertrophic gene expression by regulating alternative splicing of the MEF2 family of transcription factors^12^; and SF3B1 coordinates fructose metabolism during pathological hypertrophy, by splice isoform switching of ketohexokinase^13^.

RNA-binding motif protein 24 (Rbm24), was identified as a major regulator of alternative splicing in heart and skeletal muscle^14^. Targeted systemic inactivation of Rbm24 in mice disrupts cardiac development and results in embryonic lethality between embryonic day 12.5 and 14.5. These Rbm24 null embryos display multiple cardiac malformations including ventricular septum defects, reduced trabeculation and compaction, and dilated atria. Interestingly, sarcomerogenesis is severely abolished in the hearts of knockout embryos. Transcriptome analysis before the onset of the phenotype of Rbm24 knockout hearts revealed aberrant splicing of 68 different genes. Several of the Rbm24-regulated spliced genes have been reported to play crucial roles in cardiac and skeletal muscle development (i.e. Naca, Fxr1, Abcc9, Slc25a3, Usp25 and Usp28)^15–20^. More recently, Liu *et al*. generated a conditional knockout model for Rbm24, to be able to circumvent the embryonic lethality and study its role in the postnatal heart^21^. They showed that postnatal cardiomyocyte-specific deletion of Rbm24 (using an αMHC-Cre) induces a progressive DCM phenotype leading to lethality well before adulthood^21^.

To further explore the function of Rbm24 in the adult heart we created a systemic Rbm24 knockout mouse model using transcription activator-like effector nucleases (TALENs). The absence of Rbm24 protein resulted in embryonic lethality around E12.5 and the null embryos recapitulated the previously described cardiac phenotype, including larger atria, reduced ventricular trabeculation and ventricular septum defects^14^. To further investigate the function of Rbm24 in the adult heart, we studied heterozygous (HET) Rbm24 mice, in which Rbm24 protein levels were reduced by approximately 40%. Isolated membrane-permeabilized cardiomyocytes showed increased sarcomere slack length, which coincided with altered relations between active and passive myofilament force and sarcomere length in Rbm24 HET compared to wildtype mice. The change in sarcomere slack length did not translate into deteriorated cardiac function or altered myocardial structure in Rbm24 HET mice, neither at baseline nor in pressure overloaded hearts after transverse aortic constriction (TAC).

## Materials and Methods

### Experimental animals

All animal studies were approved by the Institutional Animal Care and Use Committee of the University of Amsterdam and carried out in compliance with the guidelines of this institution and the Directive 2010/63/EU of the European Parliament.

### Generation of Rbm24 KO mice

**Figure 1:**
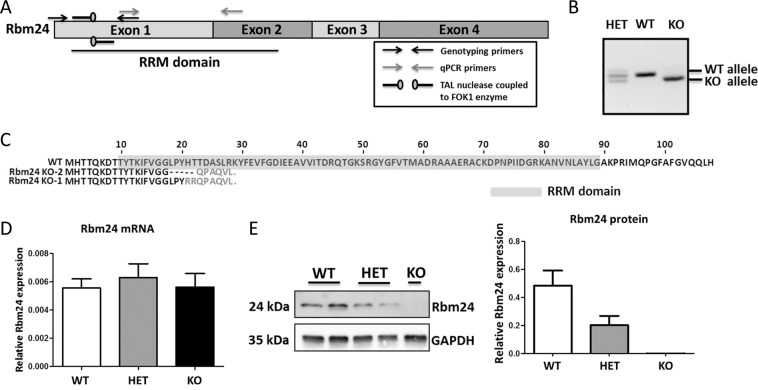
Generation of the Rbm24 knockout mouse. (**A**) Schematic representation of the Rbm24 mRNA transcript with the location of the RRM domain depicted. Black arrows indicate genotyping primers, grey arrows indicate qRT-PCR primers, and pins indicate TALEN-mediated double-stranded break location. (**B**) Genotyping of E11.5 embryos indicates correct targeting of the Rbm24 allele. (**C**) TALEN-mediated deletions within Rbm24 leads to a truncated Rbm24 protein in two independent mouse lines. Amino acids highlighted in grey indicate the RRM domain. (**D**) qRT-PCR shows that Rbm24 mRNA expression levels, corrected for GAPDH, are equal between genotypes (n = 3 per group) in hearts of E11.5 embryos. (**E**) Left panel: Western blot analysis on E11.5 hearts indicate that protein expression is reduced to 50% in Rbm24 HET (n = 2) embryos and absent in Rbm24 KO (n = 1) embryos compared to wild-types (n = 2). Right panel: corresponding quantification of Rbm24 protein levels corrected for GAPDH.

For the generation of the Rbm24 knockout mouse line we made use of the TALEN technology (transcription activator-like (TAL) effectors), described in more detail by Cermak *et al*.^22^. An upstream and downstream TALEN were designed by Polygene AG (Rumlang, Switserland) and incorporated into expression plasmids to cleave specifically within exon 1 of Rbm24. Specifically, the designed TALEN pair recognizes the following underlined sequences, resulting in the deletion of nucleotides between these two sequences (the TALEN cut region, indicated in italics):

5′-TCTTCGTGGGAGGGCTGC*CCTACCACACCACCGA*CGCCAGCCTGCGCAAGTA-3′ The expression plasmids express the TAL-Fok1 protein and contain a T7 promoter for *in vitro* RNA synthesis. Using the mMachine mMessage® T7 Transcription Ultra kit (Ambion), a poly(A) tail containing mRNA was generated from the TALEN expression plasmids following the manufacturer’s instructions. Knockout mice were generated by microinjections of the two TAL-Fok-1 mRNAs (2.5 ng/µl) into oocytes of pseudopregnant female FVB/N mice. Injected oocytes were grown to blastocyst stage and implanted back into foster mothers. About 10% of the offspring was successfully targeted, as analyzed by PCR from tissue-derived DNA. For genotyping primers see Supplemental Table 1. Two targeted lines were bred on FVB/N background for at least 6 generations. Sanger sequencing of the targeted locus revealed that in the two lines, 8 and 28 nucleotides respectively, were deleted from the Rbm24 gene, resulting in a frameshift, a premature stop codon and likely a truncated and instable Rbm24 protein (Fig. 1C). We were technically not able to detect this truncated fragment since the Rbm24 antibody we used was raised against an epitope situated in the C-terminal region of Rbm24. All experiments were performed with the KO mouse line 2, lacking 8 nucleotides in the RRM domain of Rbm24.

### Echocardiography

For the functional measurements of the adult heterozygous Rbm24 hearts, mice were subjected to transthoracic echocardiography using a Vevo 770 Ultrasound Scanner (Visual Sonics) equipped with a 30-MHz linear array transducer, under sedation with a gas mixture of O_2_ and 2.5% isoflurane. M-mode tracings in parasternal short axis view were used to measure LV internal diameter (LVID;s/d) and LV anterior wall thickness (LVAW;s/d) at end-systole and end-diastole. From the LV diameters the % of fractional shortening (%FS) was calculated using the following formula: ((LV end-diastolic diameter - LV end-systolic diameter) / LV end-diastolic diameter) x 100%.

### Transverse aorta constriction (TAC)

TAC surgery was performed on 8 weeks old male WT and Rbm24 HET mice as described before^23^ to induce sustained left ventricular pressure overload. In short, mice were sedated with 4% isoflurane and intubated for mechanical ventilation with a gas mixture of O_2_ and 2.5% isoflurane to maintain anesthesia. Thoracotomy was performed above the first rib just lateral of the sternum. The aortic arch was constricted (6-0 silk suture) together with a 27 G (Ø 0.42 mm) needle between the truncus brachiocephalicus and the arteria carotis communis sinistra. Immediately after constriction the needle was removed to restore blood flow. The sham surgery was performed identically, but without the aortic ligation. As analgesia 0.05 mg/kg carprofen was injected subcutaneously prior to surgery and at least the first two days post-surgery. Five weeks after TAC or sham surgery, mice were euthanized under anesthesia through cervical dislocation. Immediately after sacrifice, hearts were injected with 1 mol/L KCl to arrest hearts in diastole, hearts were taken out and weighed. Hearts were transversely cut and the upper half was fixated in 4% paraformaldehyde for histologic analysis and the lower half was snap-frozen in liquid nitrogen and stored at −80 °C for RNA/protein analysis and force-calcium measurements. The group sizes were as follows: 4 WT sham, 6 WT TAC, 5 HET sham, 12 HET TAC.

### RNA isolation, qRT-PCR and RT-PCR

Total RNA from embryonic hearts was isolated using TRI Reagent according to the manufacturers protocol (Sigma-Aldrich) and gentle disruption of the tissue by hand using a small pestle. Total RNA from adult LV tissue was isolated using TRI Reagent and mechanical disruption of the tissue using the MagNA Lyser and MagNA Lyser beads (Roche Diagnostics). For reverse transcription 300 ng of embryonic total RNA and 500 ng of adult total RNA was used in the Superscript II system (Invitrogen) using Oligo-dT and random hexamers as primers. The expression of different genes was determined by qRT-PCR using the Lightcycler480 Real-Time PCR system (Roche Diagnostics). Analysis of qPCR data was performed using LinRegPCR analysis software^24^. RT-PCR was used to evaluate alternative splicing changes of different genes in embryonic hearts and adult ventricular tissue with 35 cycles for the PCR. The identities of all PCR products were confirmed by Sanger sequencing. For used primers see Supplemental Table 1.

### Protein isolation and Western blotting

Protein extracts from embryonic and adult hearts were obtained by mechanical disruption of the tissue using the MagNA Lyser and MagNA Lyser beads (Roche Diagnostics) in RIPA buffer (50 mM Tris-HCl, 150 mM NaCl, 1% NP-40, 0.2% sodium deoxycholate, 0.1% SDS) supplemented with protease inhibitor cocktail (Sigma) whereafter the lysates were centrifuged at 10.000xg for 15 min at 4 °C to spin down the cell debris. Total protein concentration was determined with the BCA Protein Assay Kit (ThermoFisher Scientific) and 30–40 ug protein was loaded for Western blotting. Denatured protein extracts were separated on 4%-20% Mini-PROTEAN TGX precast gels and transferred to PVDF membranes using the Trans-Blot Turbo Transfer System (all Bio-Rad). Western blots were developed with ECL prime Western blotting detection reagent (AmerSham Biosciences) Using the ImageQuant LAS4000 (GE Healthcare Europe) images were acquired. Analysis of Western blots was performed using Image J software. The primary antibodies used for protein detection were Rbm24 (Sigma-Aldrich SAB2104677, dilution 1:1000) and GAPDH (Santa Cruz, SC365062, dilution 1:1000). Titin isoform analysis was performed after separating protein lysates on a 1% agarose gel^25^. Myofilament protein phosphorylation was analyzed using Phos-tag analysis (cardiac troponin I, cTnI) and staining of gels with SYPRO Ruby and ProQ Diamond Phosphoprotein stain as described previously^26^.

### Histology

For histological analysis, embryos were fixed for 1–2 hours in 4% paraformaldehyde and adult hearts were fixed overnight in 4% paraformaldehyde. After fixation, tissues were dehydrated in graded alcohol solutions and embedded in paraffin. Sections of embryos and hearts were cut at 7 µm and 5 µm respectively and stained with heamatoxilyn and eosin solutions or Sirius Red as previously described^27^. To determine the amount of fibrosis, 5 pictures of the Sirius Red stained sections per LV were taken with a light microscope (20x magnification). From these pictures, the Sirius Red positive area was automatically calculated as a percentage of the total tissue area using an in house made quantification macro in ImagePro 6.2. This macro provided the total amount of tissue pixels and the Sirius Red positive pixels per picture by manual threshold settings. Perivascular fibrosis was manually omitted from the pictures.

### ACTN2 immunohistochemistry

Sections of 5 μm were deparaffinized and rehydrated in a series of Xylol and ethanol. Antigens were retrieved by boiling the sections in Urea buffer (5% Urea, 100 mM Tris, pH 10.4) using a microwave. Sections were then permeabilized and blocked in 5% goat serum in 0.1% Triton x-100 in PBS for 1 hr at RT in a humidity chamber. Incubation with the primary antibody (Rabbit-anti-ACTN2, 1:250, Sigma HPA008315) in 5% goat serum in 0.1% Triton x-100 in PBS was done overnight at 4 °C. Alexa Fluor 594 conjugated antibodies were used as secondary antibodies, and DAPI was used to visualize nuclei. Pictures were taken on a Leica SP8 confocal microscope (Leica Microsystems).

### Isometric force measurements in membrane-permeabilized cardiomyocytes

Snap-frozen LV tissue from WT and HET adult Rbm24 mice was defrosted in cold relaxing solution and mechanically isolated as described^28,29^. After isolation the cells were incubated for 5 min in relaxing solution (1 mM free Mg, 100 mM KCl, 2 mM EGTA, 4 mM Mg-ATP and 10 mM imidazole; pH 7.0) containing 0.2% (v/v) Triton X-100 to remove the membranes. Thereafter, cells were washed in relaxing solution and a single cardiomyocyte was glued between a force transducer (SensoNor) and a piezoelectric motor (Physike Instrumente) with a silicon adhesive (Dow Corning)^28,29^. All measurements were carried out at 15 °C, and the composition of the relaxing and activating solutions was described previously^29^. Briefly, the isometric force measurements were performed at maximal and submaximal [Ca^2+^] (ranging from 1 to 30 μmol/L) and carried out at various sarcomere lengths (SL) ranging from 1.8 μm to 2.2 μm for WT cells and 1.9 μm to 2.3 μm for Rbm24 HET cells. After this, the cell was shortened by 30% of its length within 1 ms in a relaxing solution to determine the passive force (F_pas_) of the cells. F_pas_ was measured at the different SLs for both WT and Rbm24 HET cells. To calculate the maximal tension (in kN/m^2^) the maximal developed force (F_max_) was determined by activating the cells at a saturating [Ca^2+^], which generates the total force (F_total_). By subtracting the F_pas_ from the F_total_, the F_max_ was calculated. Maximal tension was determined by normalizing F_max_ to the cross-sectional area of the cells. The force- Ca^2+^ relations were fitted to a modified Hill equation^30^ where myofilament Ca^2+^-sensitivity was designated as the EC_50,_ indicating the [Ca^2+^] at which half of F_max_ was reached. The difference in EC_50_ at sarcomere lengths of 1.8/1.9 μm and 2.2/2.3 μm (ΔEC_50_) was determined by the length-dependent increase in myofilament Ca^2+^-sensitivity upon an increase in sarcomere length.

### Statistical analysis

All data are shown as mean ± SEM and sample size (n) is mentioned in the figure legends. The two-tailed Student’s t-test was used to determine statistical significance between two groups. In multiple group experiments, with two independent variables, samples were compared using two-way analysis of variance (ANOVA). When a significant interaction between the two main factors was observed, one-way ANOVA with a Tukey’s post-hoc test was reported. For the isometric force measurements an unpaired t-test was used to calculate (ΔEC_50_) significance and a two-way ANOVA combined with Holm-Sidak’s multiple comparisons test was used to calculate F_pas_ significance. Significance was accepted when *P* < 0.05.

## Results

### Generation of the Rbm24 knockout mouse

To study the function of Rbm24 in the heart, we generated Rbm24 knockout mice by targeted disruption of the Rbm24 gene using TALENs. The protein-coding region of Rbm24 encompasses 4 exons, of which exon 1 contains the start of the RNA recognition motif (RRM), and we designed the TALEN pair in this region (Fig. 1A). Microinjections of the two TAL-FokI mRNAs into blastocysts generated chimeric mice, which transmitted the mutant Rbm24 allele through the germline (Fig. 1B). Sanger sequencing of the mutant allele revealed a deletion of 8 nucleotides in exon 1, likely resulting in a truncated Rbm24 protein (Fig. 1C). This small deletion did however not affect the stability of the transcript, as we observed similar expression of Rbm24 mRNA in wildtype (WT), heterozygous (HET) and knockout (KO) hearts, indicating that the mutant transcript is not subjected to nonsense mediated decay (Fig. 1D). The expression of Rbm24 protein was completely absent in Rbm24 KO hearts (E11.5), and reduced to approximately 50% in the heterozygotes (Fig. 1E). Genotyping of offspring from heterozygous intercrosses in the isogenic FVB/N background yielded WT and Rbm24 HET mice in an approximate 1:2 ratio, but no Rbm24 KO mice, indicating that the homozygous deletion resulted in embryonic lethality.

### Characterization of the Rbm24 KO embryos

**Figure 2:**
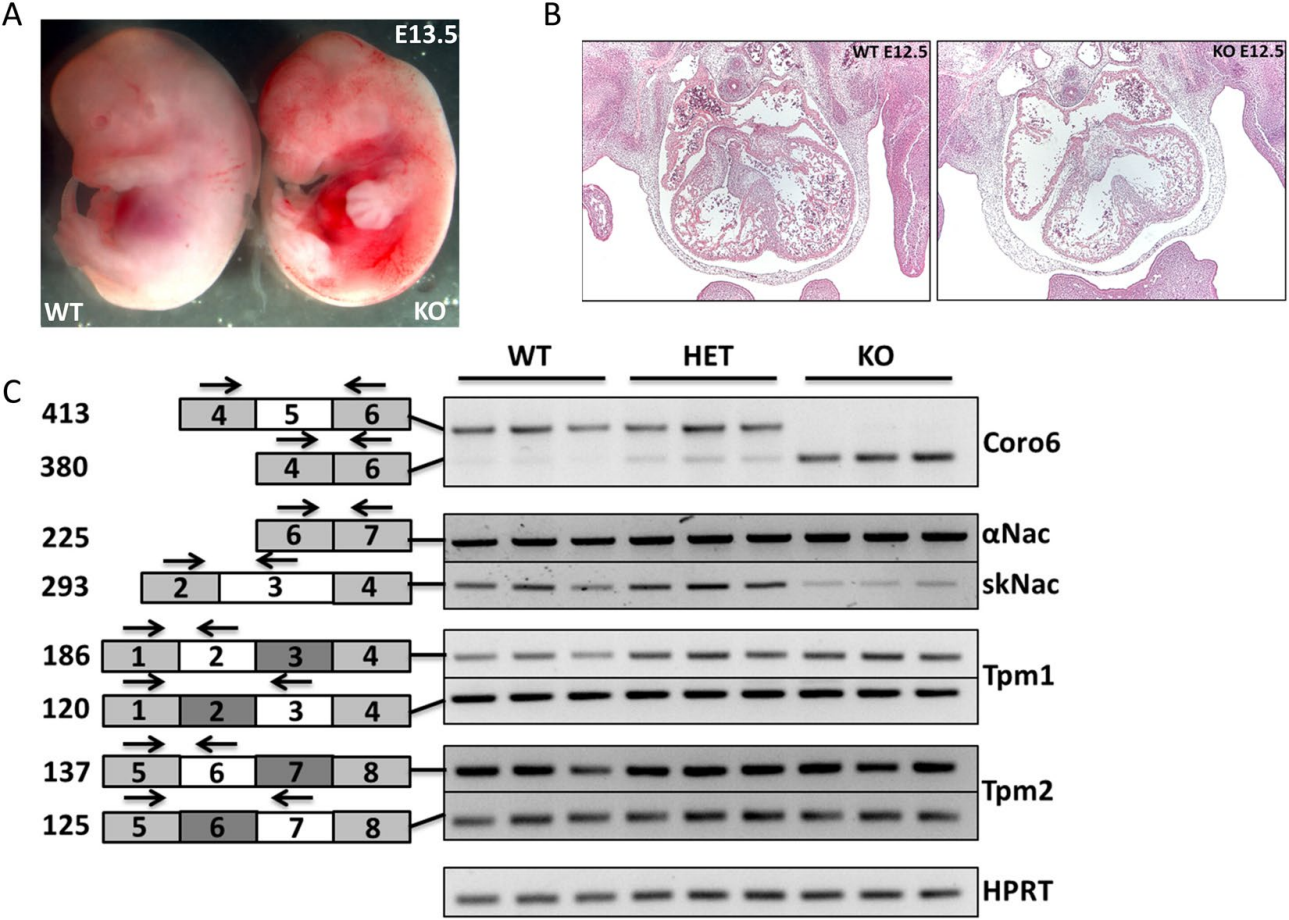
Deletion of Rbm24 leads to embryonic lethality around E12.5, disrupts cardiac development and impairs alternative splicing of multiple embryonic genes. (**A**) Example of E13.5 WT and Rbm24 KO embryo. (**B**) H&E stained sections of E12.5 WT and Rbm24 KO embryonic hearts displaying enlarged atria and reduced ventricular trabeculation in the Rbm24 KO. (**C**) Validation of alternative splicing in E11.5 Rbm24 WT, HET and KO embryos (n = 3 per group) by RT-PCR shows that alternative splicing of Coro6 and skNac is affected in the Rbm24 KO hearts. White and dark grey rectangles on the left side of the gel represent the specific exons that undergo alternative splicing. Black arrows indicate primer positions and numbers represent corresponding sizes (in base pairs) of the RT-PCR products. HPRT was used as a loading control.

To pinpoint the time of death of the Rbm24 KO embryos, we collected embryos from timed pregnancies of Rbm24 heterozygous intercrosses. Rbm24 KO embryos were present at approximate Mendelian ratios at E11.5, but around E13.5 most Rbm24 KO embryos were dead (Supplemental Table 2, Fig. 2A). Histological analysis of transverse sections of mutant embryos at E12.5 demonstrated that Rbm24 KO embryos had larger atria and reduced trabeculation of the ventricles compared to WT embryos (Fig. 2B). We next assessed alternative splicing changes in the hearts of Rbm24 KO embryos of genes involved in cardiac development and sarcomerogenesis, previously identified by Yang *et al*.^14^. In line with that study, RT-PCR analysis in E11.5 hearts revealed increased skipping of exon 5 within Coro6 and reduced inclusion of exon 3 in the muscle-specific isoform of Naca (skNac) upon deletion of Rbm24. Other previously identified Rbm24-regulated targets such as tropomyosin 1 (Tpm1) and tropomyosin 2 (Tpm2)^14^ did not show changes in alternative splicing in the embryonic Rbm24 KO hearts (Fig. 2C). In conclusion, the global Rbm24 KO mouse line that we generated, recapitulated the cardiac phenotype of the Rbm24 KO mouse line that was generated by the Braun lab^14^.

### Characterization of adult Rbm24 HET mice

**Figure 3:**
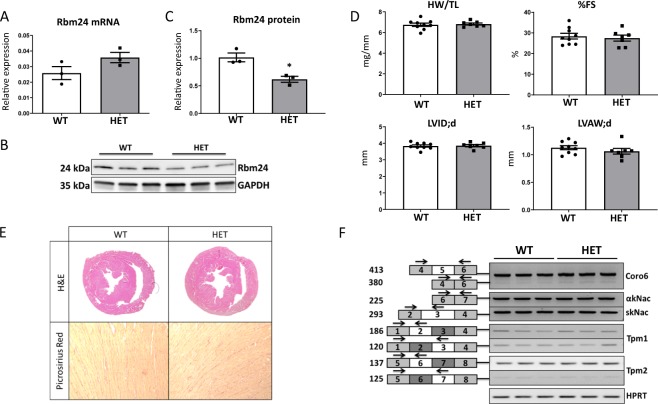
Cardiac structure, performance and alternative splicing are not altered in adult Rbm24 HET mice. (**A**) qRT-PCR shows that cardiac Rbm24 mRNA expression levels are equal between genotypes (n = 3/group). Data is corrected for GAPDH. (**B**) Western blot analysis indicates that protein expression is reduced in Rbm24 HET hearts (n = 3/group). (**C**) Quantification of Rbm24 protein levels corrected for GAPDH as shown in panel B. (**D**) Heart weight/tibia length (HW/TL) ratio is unaltered and echocardiography of WT (n = 9) and Rbm24 HET (n = 7) mice shows no difference in percentage of fractional shortening (%FS), left ventricular internal diameter in diastole (LVID;d) and left ventricular anterior wall thickness in diastole (LVAW;d) between genotypes. (**E**) Upper panel: H&E stainings reveal comparable cardiac geometry between WT and Rbm24 HET mice. Lower panel: Picrosirius Red stainings do not indicate differences in fibrosis in the hearts of WT and Rbm24 HET mice. (**F**) RT-PCR results of previously described Rbm24 target genes^14^ in hearts of WT (n = 3) and Rbm24 HET (n = 3) mice shows that alternative splicing is not altered.

To assess the potential role of Rbm24 in adult hearts, we investigated adult Rbm24 HET mice and their WT littermates in terms of cardiac structure and function, and changes in alternative splicing. In line with the embryonic Rbm24 HET hearts, we did not observe a decrease in Rbm24 mRNA levels in the adult HET hearts (Fig. 3A). However, in the hearts of adult HET mice, Rbm24 protein expression was 40% downregulated compared to WT hearts (Fig. 3B,C). Echocardiographic measurements on adult WT and Rbm24 HET mice revealed no changes in cardiac performance; as the percentage of fractional shortening (%FS), left ventricular internal diameter in diastole (LVID;d) and left ventricular anterior wall thickness in diastole (LVAW;d) were comparable between HET and WT mice (Fig. 3D). We performed H&E, Picrosirius Red stainings and α-actinin immunohistochemistry on hearts of Rbm24 HET mice, but they did not show gross abnormalities in cardiac structure, fibrosis or sarcomere organization compared to WT hearts (Fig. 3E and Supplemental Fig. 1). In addition, mRNA expression of the myofilament proteins Acta1, Actn2, Tnnt2 and Myoz2 were not significantly different between WT and Rbm24 HET hearts (Supplemental Fig. 2a). Finally, alternative splicing analysis in the Rbm24 HET hearts did not reveal aberrant splicing of the following previously identified embryonic splicing targets of Rbm24 (i.e. Coro6, Naca, Tpm1 and Tpm2, TnnT2, Ppp3ca, Cald1, Epb4, Ndgr4, Itgb1, Cox7a2l, Clta) (Fig. 3F and Supplemental Fig. 2b).

### Increased sarcomere slack length in adult Rbm24 HET cardiomyocytes

**Figure 4:**
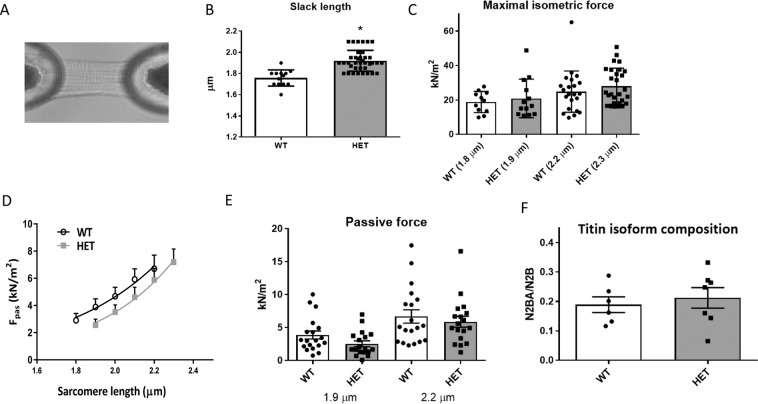
Maximal and passive myofilament force in WT and Rbm24 HET cardiomyocytes. A. Skinned cardiomyocytes, isolated from adult left ventricular tissue were glued between a force transducer and a piezoelectric motor. Scale bar = 1 μm. (**B**) Sarcomeric length at rest (slack length) is higher in Rbm24 HET cells. From WT hearts (n = 5) a total of 19 cardiomyocytes were measured, and from Rbm24 HET hearts (n = 5), 16 cardiomyocytes were analyzed. P < 0.0001 in unpaired t-test. (**C**) Maximal force did not differ between WT and Rbm24 HET groups. (**D**) Passive force generated over the entire range of sarcomere lengths (SL) was lower in Rbm24 HET cells compared to WT. For WT heart (n = 5), n = 19 cardiomyocytes were measured, for the Rbm24 HET hearts (n = 5), n = 18 cardiomyocytes were used. P < 0.05 in 2-way ANOVA. (**E**) Passive force at 1.9 and 2.2 μm did not differ between Rbm24 HET and WT. (**F**) Titin isoform composition was similar in Rbm24 HETs and WT hearts.

**Figure 5:**
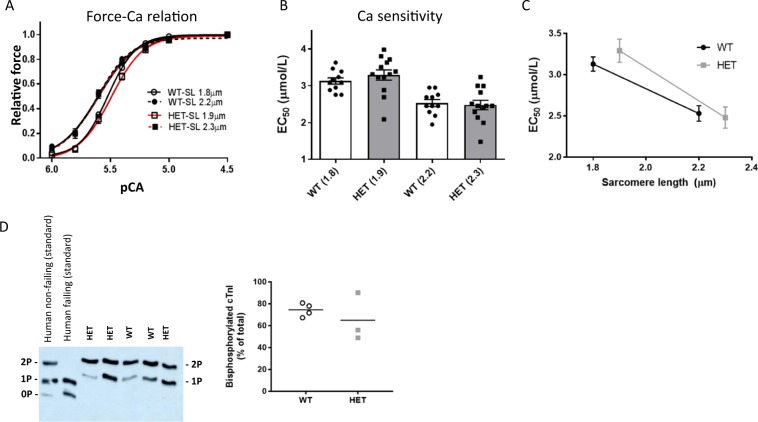
Force-calcium relations in Rbm24 HET and WT cardiomyocytes. (**A**) Isometric force plotted as a function of calcium concentration of the activating solution (pCa). The force-calcium relations at low and high sarcomere lengths are similar for both groups. For WT hearts (n = 5), 19 cardiomyocytes were measured, for the Rbm24 HET hearts (n = 5), 16 cardiomyocytes were used. (**B**) Likewise, Ca^2+^ sensitivity, depicted as EC_50_, did not differ between both groups. (**C**) EC_50_ plotted versus sarcomere length illustrates that the relation is shifted to the right in the Rbm24 HETs. (**D**) Phos-tag analysis of troponin I (cTnI) phosphorylation showed mono- and bisphophorylated forms in the Rbm24 HET and WT hearts. Human cardiac samples are included on the blot as standards to illustrate the non (0 P), mono (1 P) and bis (2 P) phosphorylated forms of cTnI. The cTnI phosphorylation pattern did not differ between WT and Rbm24 HET samples.

Guided by the publication of Yang et al^14^., who revealed that Rbm24 is required for sarcomerogenesis, we wondered whether there are functional differences in cardiomyocytes after loss of 40% Rbm24 protein levels. Therefore, we isolated cardiomyocytes from 12 weeks old WT and Rbm24 HET mice and subjected them to force-Ca^2+^ measurements, in which a membrane-permeabilized cardiomyocyte is glued between a force transducer and a piezoelectric motor (Fig. 4A). We performed these analyses on cardiomyocytes from 5 Rbm24 HET hearts and 5 WT hearts, and measured 2–5 cells per heart. The slack length, which is represented by the length of the cardiomyocyte in a resting solution, was significantly higher in Rbm24 HET cardiomyocytes compared to WT cardiomyocytes (1.9 µm vs 1.8 µm, p < 0.0001, Fig. 4B). Maximal force development did not differ between Rbm24 HET and WT cells (Fig. 4C), and did increase with increasing sarcomere length, in line with length-dependent activation of myofilaments. The relation between passive force (generated by the myocyte) and sarcomere length was shifted downwards in Rbm24 HET cardiomyocytes compared to WT cells (Fig. 4D), although values at individual sarcomere lengths did not significantly differ between groups (Fig. 4E shows values at 1.9 and 2.2 µm sarcomere length). There was no difference in titin isoform composition between HET and WT (Fig. 4F; Supplemental Fig. 3). To assess whether reduced amounts of Rbm24 protein affect Ca^2+^-sensitivity of the myofilaments, we compared the relation between force and pCa (representing the –log(10) of the Ca^2+^ concentration) in WT and Rbm24 HET cells. In Fig. 5A we plotted the isometric force as a function of the Ca^2+^-concentration, both for the low sarcomere length (1.8–1.9 µm) and for stretched sarcomeres (2.2–2.3 µm). While Rbm24 HET cells display rather similar force-pCa^2+^ relations and Ca^2+^-sensitivity (i.e. EC_50_; Fig. 5B) as the WT cells both at low and stretched sarcomere length, the relation between Ca^2+^-sensitivity and sarcomere length is different between the groups (Fig. 5C) and may be explained by the difference in sarcomere slack length. Analysis of protein phosphorylation using Phos-Tag (Fig. 5D) and ProQ Diamond stain (Supplemental Fig. 4) showed a trend towards lower phosphorylation of several myofilament proteins in Rbm24 HETs compared to WT, but only reached statistical significance for cardiac myosin-binding protein C (cMyBP-C). Reduced phosphorylation of MyBP-C, a target of protein kinase A, is in line with previous observations in cardiac disease, and may be related to desensitization/downregulation of the beta-adrenergic receptor pathway.

Overall, these findings indicate that reduced levels of Rbm24 increases sarcomere slack length and shifts the relation between myofilament force development and sarcomere length. These small changes did not appear to affect cardiac function at baseline, as measured by echocardiography (Fig. 3D).

### Cardiac function after sustained pressure overload is not different between WT and Rbm24 HET mice

**Figure 6:**
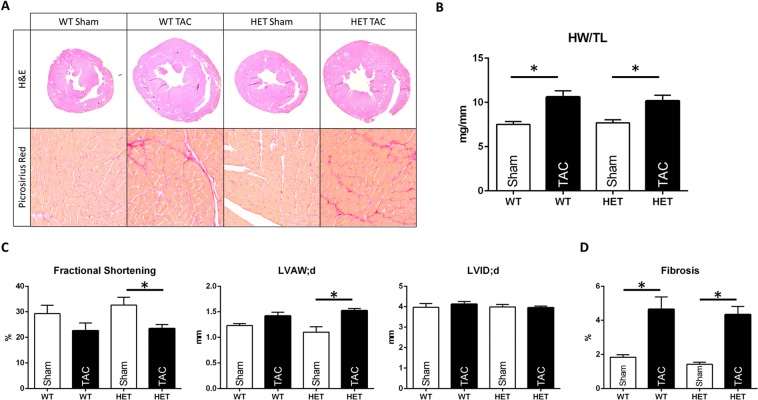
Transverse aortic contriction (TAC) in Rbm24 heterozygotes. (**A**) Upper panel: H&E stainings on cardiac sections of sham- and TAC- operated WT and Rbm24 HET mice show no differences in hypertrophy or LV dilation, 5 weeks after surgery. Lower panel: Picrosirius Red stainings do not indicate differences in fibrosis in the hearts of WT and Rbm24 HET mice after TAC surgery. (**B**) Heart weight/tibia length ratio (HW/TL) was equally increased in both WT and Rbm24 HET mice. (**C**) Echocardiography on sham-operated (n = 4) and TAC operated (n = 6) WT mice and sham-operated (n = 5) and TAC-operated Rbm24 HET mice (n = 12). No differences were observed in the percentage of fractional shortening, left ventricular internal diameter in diastole (LVID;d) and left ventricular anterior wall thickness in diastole (LVAW;d) between WT and Rbm24 HET hearts after TAC surgery. (**D**) Quantification of Picrosirius Red stainings revealed a statistically significant increase in fibrosis in both genotypes, but no differences were observed between WT and Rbm24 HET hearts (2-way ANOVA).

To investigate whether the reduction in Rbm24 protein in HET mice affects cardiac structure or function after induction of stress to the heart, we performed thoracic aortic constrictions (TAC). Five weeks after TAC or sham surgery, echocardiography was performed and mice were sacrificed, hearts excised and cardiac structure was analyzed. H&E stainings on TAC- and Sham-operated hearts revealed that Rbm24 heterozygosity does not affect gross cardiac morphology (Fig. 6A). Both WT and Rbm24 HET mice showed a significant increase in heart weight/tibia length ratio (HW/TL) 5 weeks after TAC, but the response to cardiac pressure overload was comparable between the two genotypes (Fig. 6B). M-mode echocardiography in these mice showed that cardiac function (as indicated by fractional shortening) was comparable between the two groups. We also did not detect differences in left ventricular internal diameters (LVID;d) or wall thickness (LVAW;d) between Rbm24 HET and WT mice (Fig. 6C). Picrosirius red stainings were performed on cardiac sections of both genotypes but did not reveal differences in the collagen deposition at baseline (Sham) or 5 weeks after TAC (Fig. 6A,D). Finally, the cardiac stress marker atrial natriuretic peptide (Nppa) was induced to a similar extent in WT and Rbm24 HET hearts after TAC (Supplemental Fig. 5). In conclusion, cardiac remodeling after sustained pressure overload was not affected in Rbm24 HET mice.

## Discussion

In this study we generated somatic Rbm24 KO mice and demonstrate that homozygous KOs are embryonically lethal. Rbm24 KO mice die around E12.5 and show disrupted cardiac development, thereby recapitulating the Rbm24 KO phenotype previously reported by Yang *et al*.^14^. In E11.5 Rbm24 KO hearts, we observed changes in alternative splicing of multiple genes (e.g. Coro6, skNac), which further corroborates the finding that Rbm24 acts as a splicing factor in the developing heart. To investigate the putative role of Rbm24 in the adult heart we made use of the Rbm24 heterozygotes (HETs), in which Rbm24 protein levels were reduced by 40%. We examined potential functional differences in cardiomyocytes of Rbm24 HET mice by measuring force-Ca^2+^ relations in isolated cardiomyocytes and reveal that reduced levels of Rbm24 affect myofilament performance, in particular by changing sarcomere slack length. The underlying mechanism for the altered sarcomere length-force relation is not known. The adult Rbm24 HET hearts were structurally normal (e.g. H&E and Sirius red), no change in titin isoform composition was observed, and echocardiography did not reveal compromised cardiac performance at baseline. Even after 5 weeks of sustained LV pressure overload, we did not observe changes in cardiac structure or performance in the Rbm24 HET mice as compared to WT controls. In conclusion, we found altered sarcomere length-force relations in membrane-permeabilized cardiomyocytes from adult HET mice expressing ~60% of WT Rbm24 protein level, but this did not coincide with altered *in vivo* cardiac function, neither at baseline, nor at 5 weeks after pressure overload of the heart.

The absence of an overt functional phenotype in Rbm24 HET hearts at baseline and after TAC, despite altered length-force relations in isolated myofilaments, may relate to several issues. First, 60% of Rbm24 protein in the heart may be sufficient to maintain proper splicing of its target proteins, as we did not observe major structural or protein changes. A second point may relate to the genetic background used in the Rbm24 KO model. It has been shown that different mouse strains can vary drastically in their susceptibility to disease^31^. In this study, we used the genetic inbred strain FVB/N, which is rather resistant to heart disease. The C57BL/6 strain however, is the most commonly used strain in cardiovascular research, because this inbred strain is more susceptible to cardiovascular phenotypes^31^. Therefore, it is well possible that the use of C57BL/6 instead of FVB/N mice, would have resulted in a cardiac phenotype after TAC upon 40% loss of Rbm24. Third, potential differences in extracellular matrix, and all proteins involved in coupling/connecting the myocytes to the matrix, may explain why different sarcomere length in isolated cells may not compromise global *in vivo* function. Fourth, we assessed myofilament calcium sensitivity at a temperature of 15 °C. We may not be able to directly translate demembranized myofilament measurements at cold temperature to the beating heart. Fifth, the fact that myofilament calcium sensitivity was assessed at steady state, whereas in the beating heart, it is in a dynamic state may also explain the *in vitro* versus *in vivo* differences.

We conclude that under the conditions in this study, the slightly altered myofilament function in Rbm24 HETs does not translate in impaired cardiac remodeling and function.

In this regard, it is relevant to mention that Rbm38, a closely related family member of Rbm24 is expressed in the heart as well. Rbm24 and Rbm38 share 68% of sequence identity, which suggests they could be genetically redundant^32^. In a recent study, we generated somatic Rbm38 KO mice to investigate its role in the heart. Homozygous loss of Rbm38 resulted in hematopoietic defects but cardiac form and function were not affected, neither at baseline, nor during pressure overload-induced cardiac remodeling^32^. In that study, it was suggested that Rbm24 compensated for the loss of Rbm38. To confirm possible functional redundancy between Rbm38 and Rbm24 it would be interesting to examine cardiac pathophysiology in compound Rbm24/Rbm38 KO mice. It is conceivable that introducing one mutant Rbm24 allele into the Rbm38 null background would lead to altered splicing and cardiac dysfunction, in line with the hearts of the Rbm24 null embryos.

Recently, Liu and colleagues generated a conditional Rbm24 knockout allele in mice^21^. Interestingly, postnatal deletion of Rbm24, specifically in cardiac myocytes (using αMHC-Cre) resulted in a disrupted sarcomere structure and a severe DCM phenotype, with all mice dying before adulthood^21^. Also in their study, Rbm24 HET mice were largely unaffected, suggesting that half the level of Rbm24 is sufficient for normal cardiac function.

Our laboratory recently demonstrated that overexpression of Rbm24 in the postnatal and adult mouse heart, by use of adeno-associated virus serotype 9 (AAV9) resulted in increased expression of Tgfβ- and extracellular matrix related genes, increased activation of cardiac fibroblasts and extensive cardiac fibrosis^33^. The observation that Rbm24 overexpression increases ECM-related gene expression is in line with the study of Poon *et al*., who showed that Rbm24 knockdown in HL-1 cardiomyocytes decreases the expression of several Tgf-β and ECM-genes, such as Timp1, Ctgf and Tgfβ2^34^. In the current study, however, loss of Rbm24 did not lead to diminished cardiac fibrosis (Fig. 6D) and we did not find decreased expression of ECM-related genes (Supplemental Fig. 5). This indicates that ~60% of the normal Rbm24 amount is still sufficient to induce a fibrotic response during cardiac remodeling.

Although RBM24 has not (yet) been identified as a clinically relevant disease gene for human DCM^7,35^, our finding of altered sarcomere slack length in Rbm24 haploinsufficient mice does indicate that it is conceivable that pathogenic variants in RBM24 underlie human cardiomyopathy in some cases. Alternatively, it is well possible that aberrant RBM24 levels or mutations in RBM24, can affect disease progression in acquired heart disease or disease penetrance in genetic forms of heart failure. Therefore, we believe further studies relating RBM24 to familial forms of DCM or heart failure progression are needed.

## Supplementary information

Supplementary information.
