# Supplementary material for: Heterozygous loss of Rbm24 in the adult mouse heart increases sarcomere slack length but does not affect function
Source: Sci Rep. 2020 May 6;10:7687. doi: 10.1038/s41598-020-64667-0 (PMC7203132; doi:10.1038/s41598-020-64667-0)
Supplement: Supplementary file 1 — Supplementary information. [file 41598_2020_64667_MOESM1_ESM.pdf]

Supplemental information

Heterozygous loss of Rbm24 in the adult mouse heart increases sarcomere slack length but does not affect function

de Groot NE[1*], van den Hoogenhof MMG[1*], Najafi A[2], van der Made I[1], van der Velden J[2], Beqqali A[1], Pinto YM[1], Creemers EE[1]

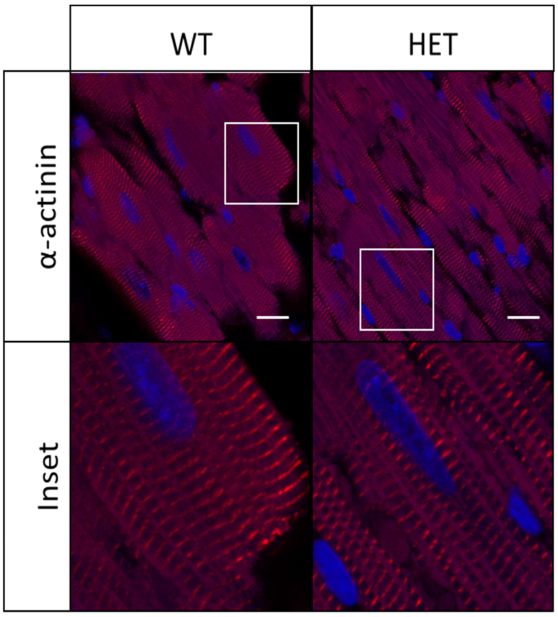

Supplemental Figure 1: α-actinin immunohistochemistry hearts of adult WT and Rbm24 HET mice.

Representative images are shown and scale bar represents 20μm.

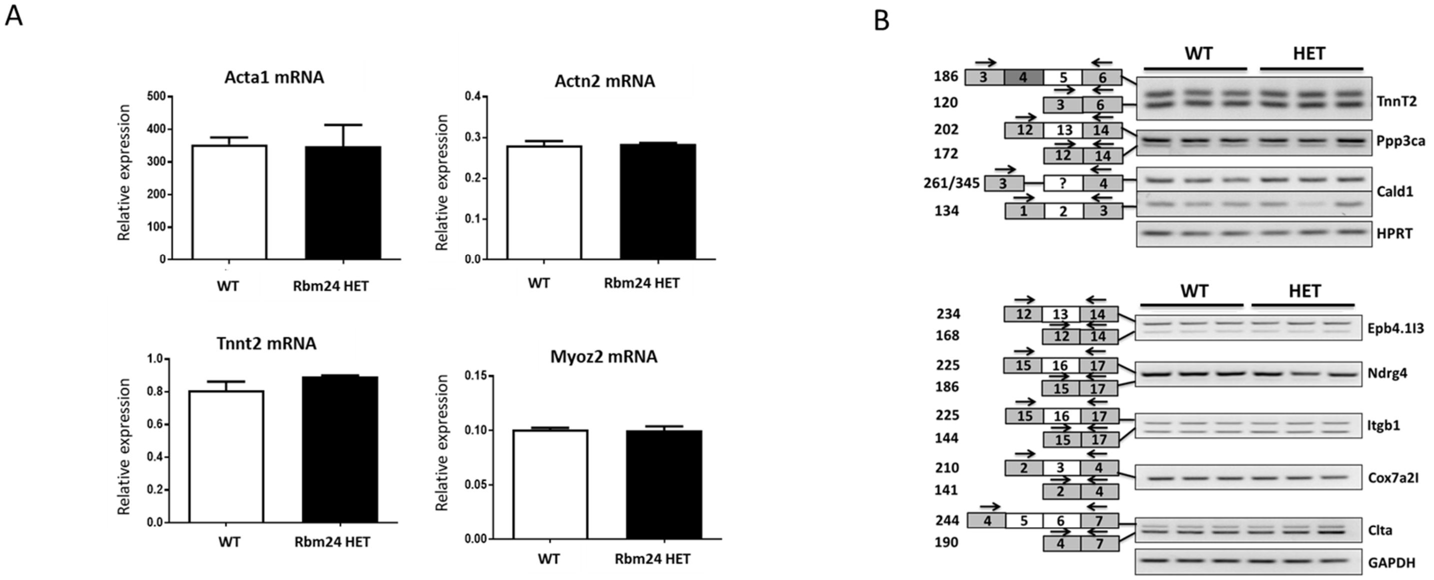

Supplemental Figure 2: Alternative splicing and mRNA expression in adult Rbm24 HET hearts. A. qRT-PCR for mRNA of Acta1, Actn2, Tnnt2, Myoz2 and Ryr2 in adult Rbm24 HET hearts (n=3) and in WT controls (n=3). Expression is normalized by GAPDH expression. Student's t-test did not reveal significant changes. **B.** RT-PCR results of previously identified Rbm24 target genes[14] in adult hearts of WT (n=3) and Rbm24 HET (n=3) mice show that alternative splicing is not affected.

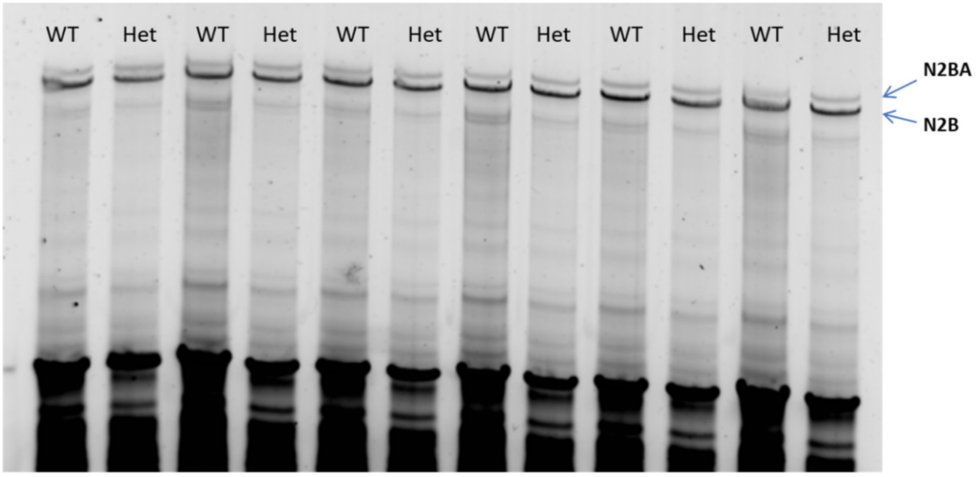

Supplemental Figure 3: Titin isoforms analysis by SDS-agarose gelelectrophoresis. SDS-agarose gel electrophoresis of protein extracts of WT and Rbm24 HET hearts shows the expression of two TTN isoforms: N2BA and N2B.

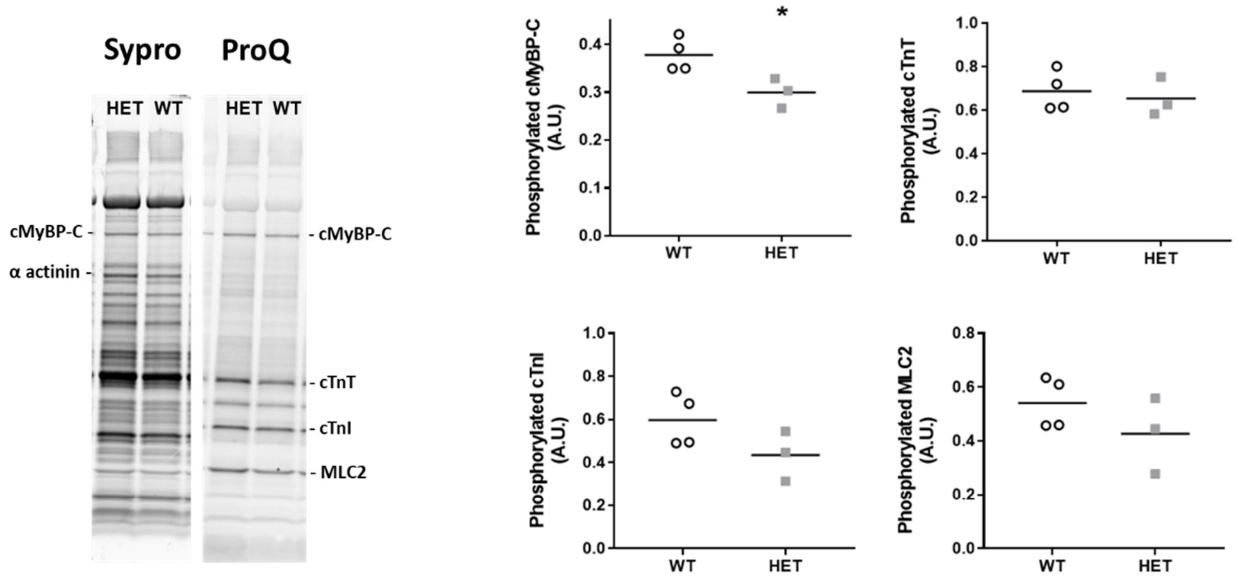

Supplemental Figure 4: Proteins separated by gel electrophoresis were stained with SYPRO Ruby and Pro-Q Diamond Phosphoprotein Stain and enables the simultaneous analyses of expression levels and phosphorylation of myofilament proteins cardiac myosin-binding protein C (cMyBP-C), cardiac troponin T (cTnT), cardiac troponin I (cTnI) and myosin light chain 2 (MLC2). 3 WT and 3 Rbm24 HET hearts were analyzed. A slightly lower phosphorylation of cMyBP-C was observed in HET compared to WT. Student's T-test *P<0.05.

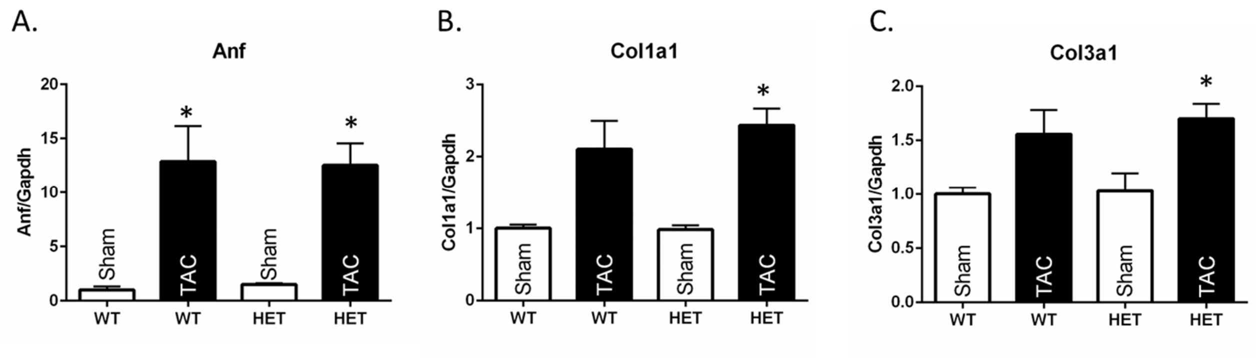

Supplemental Figure 5: qRT-PCR analysis of WT and Rbm24 HET hearts after 5 weeks of TAC. qRT-PCR analysis of Anf, Col1a1, and Col3a1 in sham-operated (n=4) and TAC- operated (n=6) WT hearts and sham-operated (n=5) and TAC-operated (n=12) Rbm24 HET hearts. * $p<0.05$ compared to sham animals of the same genotype. No significant interactions were found between the intervention and the genotype by 2-way ANOVA.

Supplemental Table 1: Primers used for genotyping, qRT-PCR and RT-PCR

Gene	Primer sequence
**Genotyping Rbm24 fw**	gcacaccacccagaaggacacg
**Genotyping Rbm24 rv**	ggtctgtgatgaccaccgct
**Rbm24 fw**	gccagcctgcgcaagtacttt
**Rbm24 rv**	gttgggatccttgcaggccctt
**skNAC fw**	tacagagcaggagttgccac
**skNAC rv**	gcagtttcagctgttatggg
**Coro6 fw**	tcatcatctggaatgtgggc
**Coro6 rv**	gtaccgaatgctactgtcac
**α-NAC fw**	tacagagcaggagttgccac
**α-NAC rv**	ctaactgtgcttgctgagac
**Col3a1 fw**	tcaaggctgaaggaaacagca
**Col3a1 rv**	gatgggtagtctcattgcc
**ANF fw**	attgacaggattggagcccagagt
**ANF rv**	tgacacaccacaagggcttaggat
**GAPDH fw**	ggtggacctcatggcctaca
**GAPDH rv**	ctctcttgctcagtgtccttgct
**HPRT fw**	cctaagatgagcgcaagttgaa
**HPRT rv**	ccacaggactagaacacctgctaa

Supplemental Table 2: Viable embryos collected from Rbm24 heterozygous intercrosses

Embryonic stage	Genotype	# of embryos
**E11.5**	WT	6 (22%)
	HET	15 (56%)
	KO	6 (22%)
**E12.5/E13.5**	WT	11 (40%)
	HET	14 (52%)
	KO	2 (7%)

Full gels

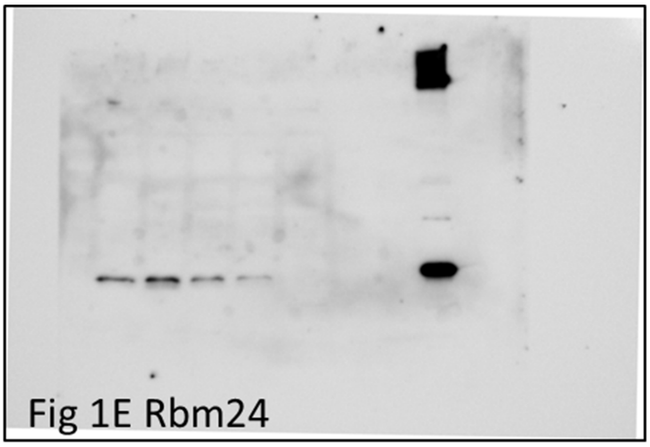
Fig 1E Rbm24

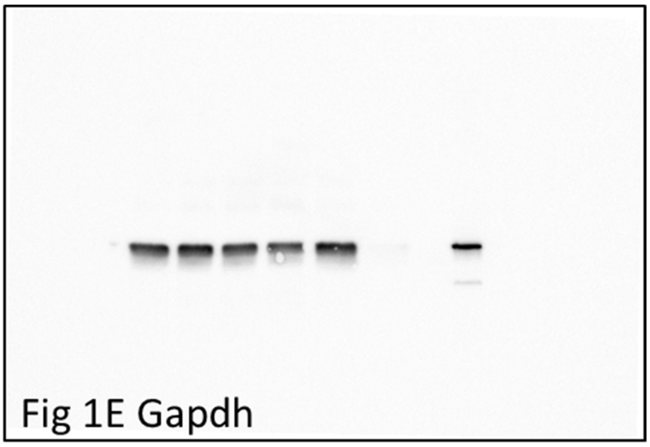
Fig 1E Gapdh

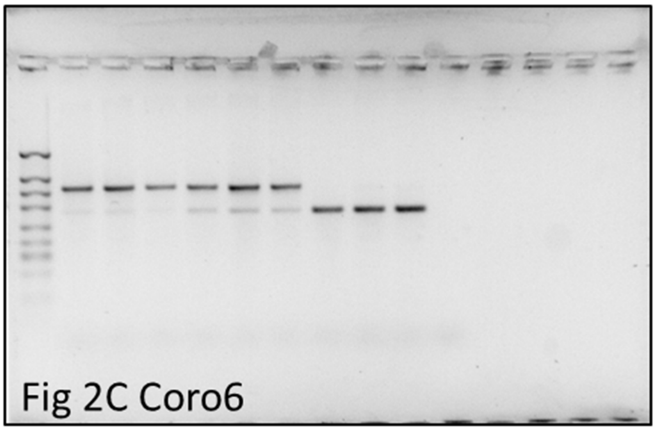
Fig 2C Coro6

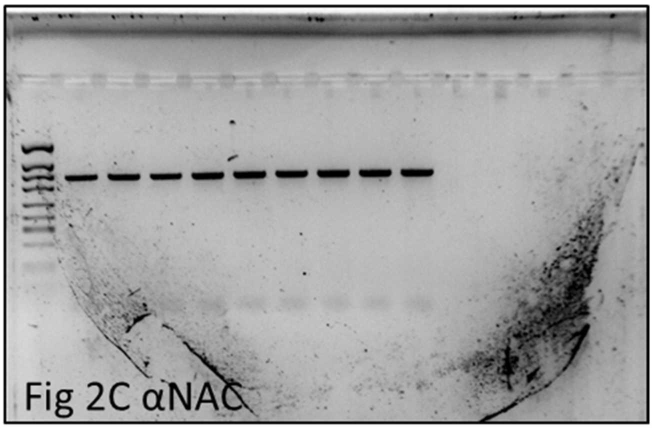
Fig 2C αNAC

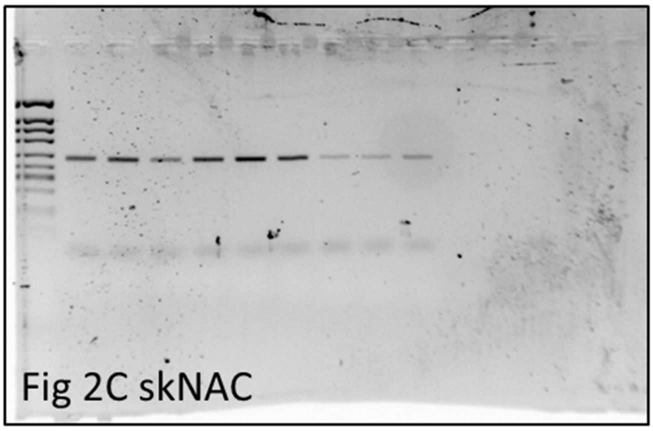
Fig 2C skNAC

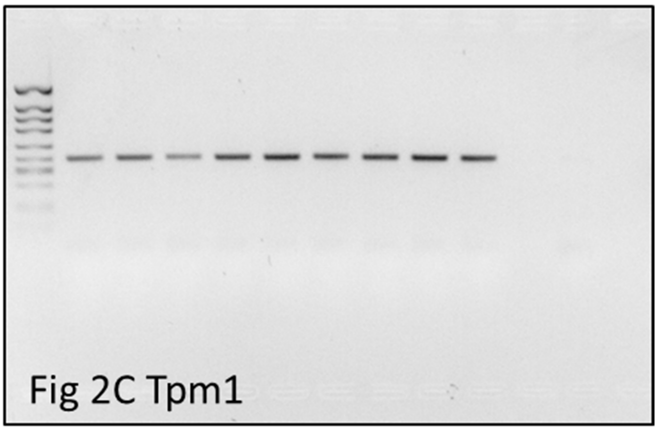
Fig 2C Tpm1

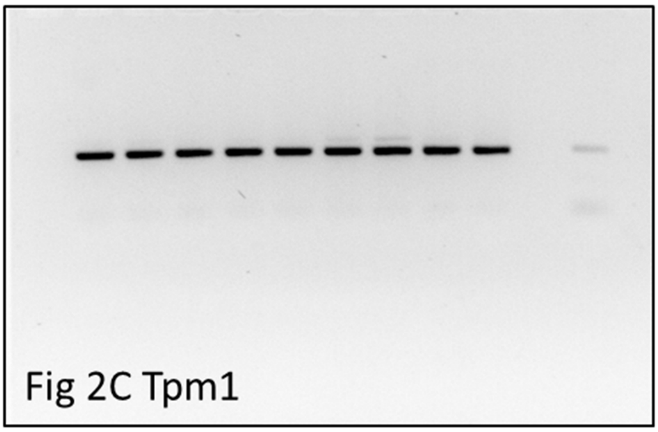
Fig 2C Tpm1

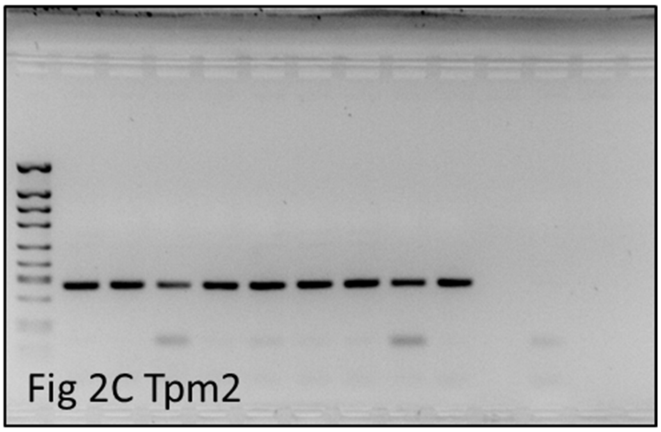
Fig 2C Tpm2

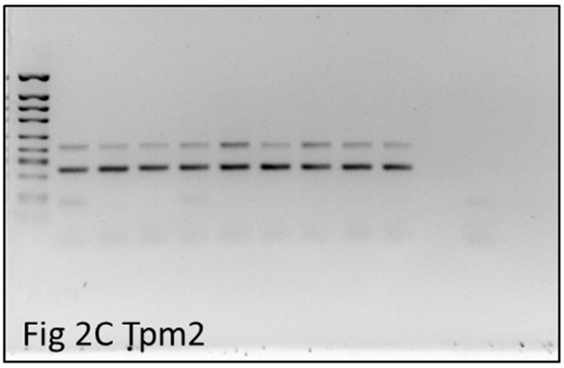
Fig 2C Tpm2

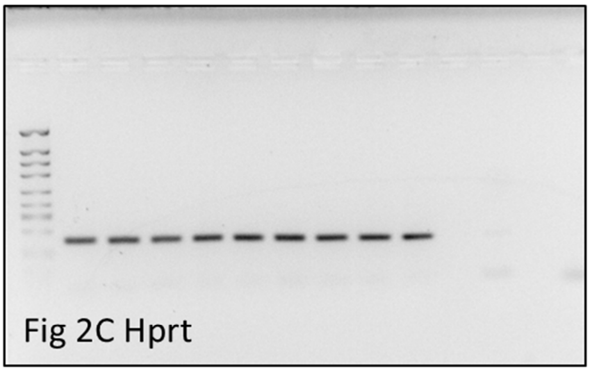
Fig 2C Hprt

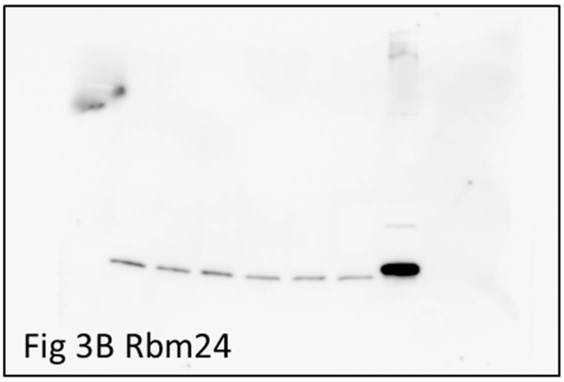
Fig 3B Rbm24

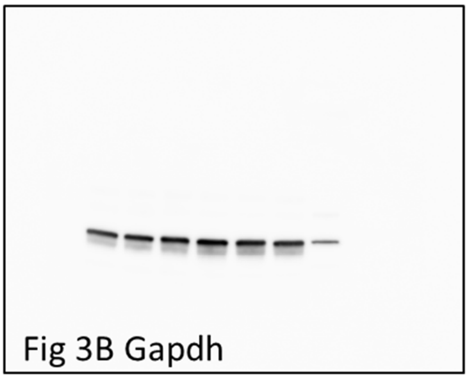
Fig 3B Gapdh

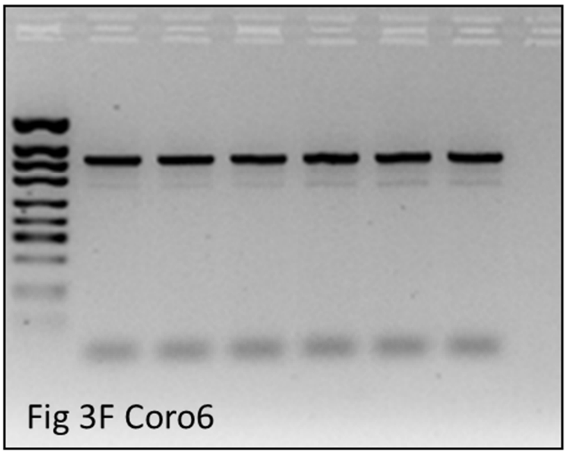
Fig 3F Coro6

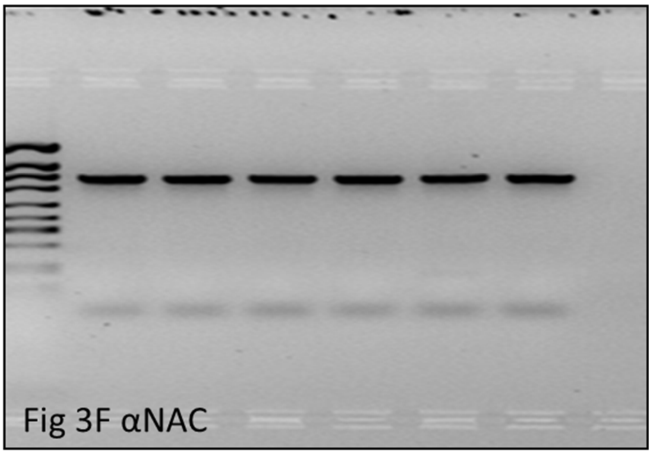
Fig 3F αNAC

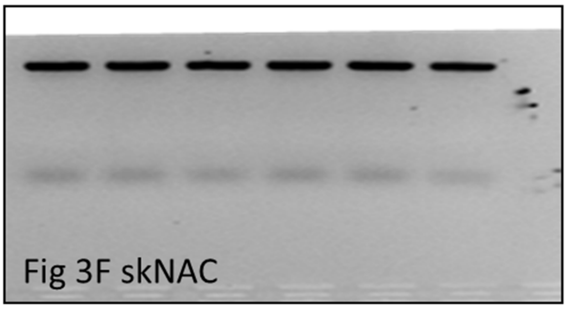
Fig 3F skNAC

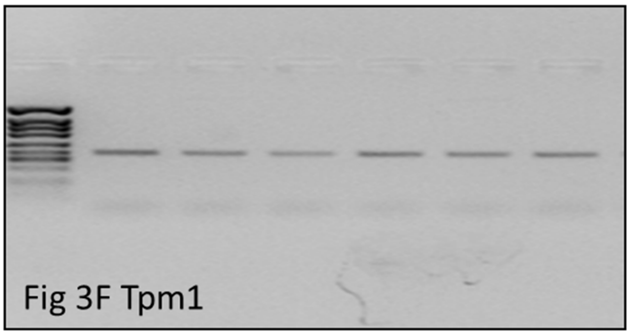
Fig 3F Tpm1

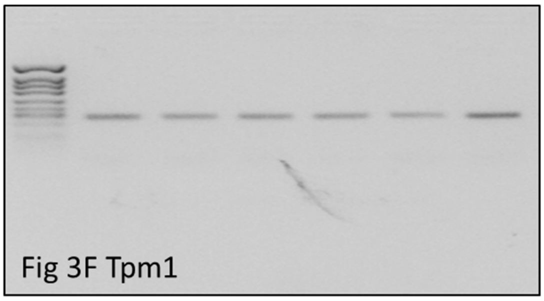
Fig 3F Tpm1

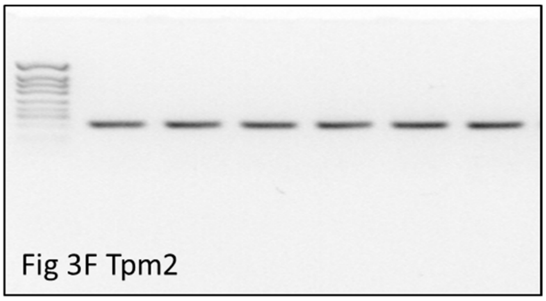
Fig 3F Tpm2

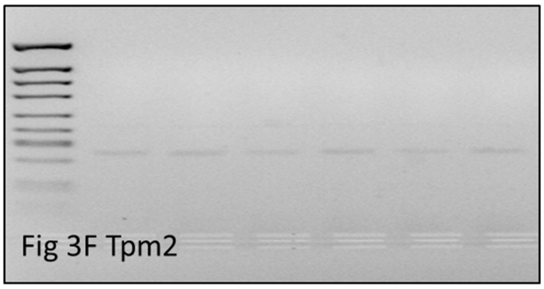
Fig 3F Tpm2

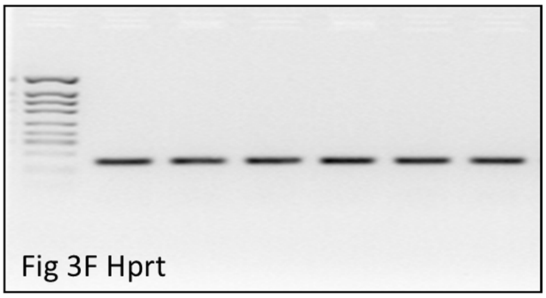
Fig 3F Hprt
